# Increased host diversity limits bacterial generalism but may promote microbe-microbe interactions

**DOI:** 10.1093/ismeco/ycaf146

**Published:** 2025-08-23

**Authors:** Iris A Holmes, José G Martínez-Fonseca, Rudolf von May, Briana A Sealey, Peter A Cerda, Maggie R Grundler, Erin P Westeen, Daniel Nondorf, Joanna G Larson, Christopher R Myers, Tory A Hendry

**Affiliations:** Cornell Institute of Host-Microbe Interactions and Disease, Cornell University, Ithaca, NY 14853, United States; Department of Microbiology, Cornell University, Ithaca, NY 14853, United States; Department of Public and Ecosystem Health, Cornell University College of Veterinary Medicine, Ithaca, NY 14853, United States; Museum of Zoology and Department of Ecology and Evolutionary Biology, University of Michigan, Ann Arbor, MI 48109, United States; School of Biological Sciences, Southern Illinois University, Carbondale, IL 62901, United States; School of Forestry, Northern Arizona University, Flagstaff, AZ 86011, United States; Museum of Zoology and Department of Ecology and Evolutionary Biology, University of Michigan, Ann Arbor, MI 48109, United States; Biology Program, California State University Channel Islands, Camarillo, CA 93012, United States; Museum of Zoology and Department of Ecology and Evolutionary Biology, University of Michigan, Ann Arbor, MI 48109, United States; Department of Integrative Biology, University of Texas, Austin, TX 78712, United States; Museum of Zoology and Department of Ecology and Evolutionary Biology, University of Michigan, Ann Arbor, MI 48109, United States; Museum of Zoology and Department of Ecology and Evolutionary Biology, University of Michigan, Ann Arbor, MI 48109, United States; Department of Environmental Science, Policy, and Management and Museum of Vertebrate Zoology, University of California, Berkeley, CA 94720, United States; Museum of Zoology and Department of Ecology and Evolutionary Biology, University of Michigan, Ann Arbor, MI 48109, United States; Department of Environmental Science, Policy, and Management and Museum of Vertebrate Zoology, University of California, Berkeley, CA 94720, United States; Museum of Zoology and Department of Ecology and Evolutionary Biology, University of Michigan, Ann Arbor, MI 48109, United States; Department of Biology, University of Virginia, 485 McCormick Road Charlottesville, VA 22904, United States; Museum of Zoology and Department of Ecology and Evolutionary Biology, University of Michigan, Ann Arbor, MI 48109, United States; Department of Biological Sciences and Museum of Biodiversity, University of Notre Dame, Notre Dame, IN 46556, United States; Cornell Institute of Host-Microbe Interactions and Disease, Cornell University, Ithaca, NY 14853, United States; Center for Advanced Computing & Laboratory of Atomic and Solid State Physics, Cornell University, Ithaca, NY 14853, United States; Cornell Institute of Host-Microbe Interactions and Disease, Cornell University, Ithaca, NY 14853, United States; Department of Microbiology, Cornell University, Ithaca, NY 14853, United States

**Keywords:** host associated microbiome, bacterial diversity, squamate reptile, cloacal microbiome, Firmicutes, Proteobacteria, bacterial host generalism

## Abstract

Host-associated bacteria vary in the number of host species they occupy. By colonizing many host species, host generalists can have disproportionate ecological impacts and should gain an evolutionary advantage when host species availability varies. However, past work has shown that many bacterial lineages are host specific. We hypothesized that constraints on bacterial host generalism will differ depending on ecological context. To test this, we assessed patterns of diversity and specialization in the cloacal microbiomes of reptile communities from the temperate zone to the tropics, a 10-fold increase in host species richness. We found that some host-specific lineages increased in richness along with their hosts, while generalist lineages did not. Generalist lineages were able to attain their highest host prevalence when host diversity was lower. In our highest diversity host communities, we found that the successful generalists, typically Proteobacteria, were disproportionately likely to co-occur with one another across evolutionarily disparate hosts. Our data indicated that bacterial lineages may adapt to the evolutionary pressures of high diversity host communities either by specializing on hosts or by forming cohorts of co-occurring bacterial lineages. Previous research across vertebrate gut microbiomes has shown that mutually beneficial relationships between bacterial lineages are widespread. Our work further supports that finding and contextualizes it within a range of host community diversity.

## Introduction

Like all organisms, bacterial lineages experience conflicting selective pressures between specialization and generalism [1]. Evolutionary theory predicts that when resources are consistently available, specialists should have a competitive advantage relative to generalists, whereas in fluctuating environments generalists have an advantage [2, 3]. In real communities, complex patterns of variation in selective pressures allow specialists and generalists to coexist [4, 5]. While most bacterial lineages are host or habitat specialists [6, 7], host-associated generalist lineages can carry outsized ecological importance as potential pathogens [8] or mutualists [7, 9] that can interact with many host species at once. Despite the ecological importance of generalist microbes, constraints on maximal host generalism in bacteria are poorly understood [10]. Here, we seek to understand whether fundamental host-bacteria incompatibilities limit bacterial host generalism, or whether host generalism may be more constrained by ecological interactions between bacterial lineages in host-associated microbial communities [11–13].

We studied ecological interactions between bacterial lineages in the functionally important cloacal microbiome of snakes and lizards. Cloacas are semi-oxygenated habitats at the terminus of the digestive, urinary, and reproductive tracts [14]. Cloacal microbiota composition can be impacted by host diet, habitat, and stress levels [15–17]. These communities can include parasites that lead to host morbidity or mortality [18–21]. The cloaca also houses bacterial lineages that inoculate the eggshell during laying and may increase host fitness by providing protection against fungal infection [22], possibly leading to vertical transmission of bacteria [23, 24]. Gut bacteria can colonize the cloacal microbiome, but bacterial lineages also colonize through mating or through contact with the external environment [15, 25, 26]. Adaptation to the cloacal habitat, competition between bacteria, and interaction with host immune processes can all structure the cloacal community [27–30]. This combination of complex bacteria-bacteria and bacteria-host interactions, and the different selective pressures provided by horizontal and vertical transmission of the bacterial lineages that make up the community, makes the cloacal microbiome an ideal site for studying the constraints on bacterial lineage host generalism.

In this paper, we assess the host generalism of our bacterial lineages in two ways. First, we quantify each lineage’s ability to associate with a broad phylogenetic range of hosts and second, the lineage’s prevalence in our samples, regardless of host species. By incorporating both perspectives, we indirectly test generalism across aspects of host ecology and physiology not captured by host phylogeny, potentially include aspects such as diet, immune factors, or habitat [31–35]. In addition, we compare the host phylogenetic generalism and host prevalence patterns of two of the most common host-associated bacterial phyla, the Proteobacteria and the Firmicutes [36–38], found in the reptile cloaca [26, 39]. In mammals, at least two common, obligately anaerobic families in the Firmicutes phylum are transmitted primarily by vertical inheritance and can be more host lineage-specific [40]. Many Proteobacteria taxa are facultatively aerobic [41] and do not show a similar tendency toward vertical inheritance [40]. It is unclear if the mechanisms maintaining host specificity in mammalian microbiota apply to lizards and snakes [42], but this evidence motivated us to investigate whether the two phyla might have distinct patterns of host occupancy in squamate reptiles as well as mammals.

We sampled a latitudinal transect of lizard and snake communities spanning a 10-fold increase in species richness to test whether host community richness structures microbial diversity or host associations [43]. We hypothesized that high-richness host communities will also have the highest richness of microbial lineages, as has been observed for some parasite communities [44, 45]. In addition, free-living generalist bacterial communities are more diverse toward the tropics in some habitats, so host-associated bacterial lineages could follow this pattern [46]. Second, we hypothesized that bacterial lineages in high host richness communities will be more host-specialized due to the challenges of adapting to greater variation in host immune defenses, diet, and other ecological factors. Finally, we hypothesized that bacteria-bacteria interactions will be strongest between host-specialist lineages. Host specialization could promote either mutualistic or competitive interactions between bacterial lineages by repeatedly bringing them together within a host species, while generalist lineages may have experienced selective pressure toward tolerating a wide range of other bacterial lineages.

## Materials and methods

### Sample collection, sequencing, and bioinformatics

We sampled cloacal microbiomes of snakes and lizards from six sampling sites, from Michigan in the northern United States to the Amazon rainforest in Peru. We divided sites into host richness categories by the number of host families present, as microbiome communities are consistently differentiated at the host family level (Table 1) [26]. Aside from our most northerly site in Michigan where we sampled only snakes, all sites had similar maximum phylogenetic breadth between hosts but varied in host family richness. This allowed us to specifically compare impacts of host species richness, not total phylogenetic breadth, on our bacterial ecology metrics. For details on host species identification and phylogeny, see supplemental methods. We sampled low host taxonomic richness sites in temperate forest habitats near Ann Arbor, MI and Girard, GA; moderate richness in tropical montane pine forest and cloud forest at Las Brisas del Mogoton and tropical dry forest at Asosoca Lake in Nicaragua; and high richness in tropical wet forest at Refugio Bartola in Nicaragua [47] and Los Amigos Biological Station in Peru (Table 1; Table S1). Further details of sampling strategy and potential caveats are available in the supplemental methods. Capture techniques were approved by University of Michigan UCUCA Protocol #PRO00006234 and protocol #I16005 issued by Georgia Southern University IACUC to Christian L. Cox.

**Table 1:** Site-specific information.

	Michigan temperate forest	Georgia temperate forest	Nicaragua high elevation	Nicaragua dry forest	Nicaragua wet forest	Peru wet forest
**Species present**	14	39	25	49	79	99
**Species sampled**	5	12	15	15	29	30
**Genera sampled**	3	10	11	12	23	24
**Families sampled**	2	5	8	7	10	7
**Maximum phylogenetic spread**	0.605	1.831	2.080	2.184	2.324	2.442
**Average lat and long of all points**	42.302, −83.663	32.5135, −81.263	13.731, −86.374	12.416, −86.653	10.973, −84.335	−12.570, −70.083
**Sampling area maximum distance (km)**	1.5	125.12	3.2	4.0	1.8	7.0
**Habitat type**	Temperate forest	Temperate forest	Upland pine forest and cloud forest	Tropical dry forest	Tropical wet forest	Tropical wet forest

Species and higher taxon richness present and sampled at each site, as well as mean latitude and longitude, maximum distance between samples within a site, and habitat type of the sampling locality.

We sampled microbiome DNA using a sterile rayon swab (MW113) inserted into the cloaca and quickly removed to collect cloacal mucosa and epithelial cells [48]. We detail our laboratory and bioinformatics approaches in the supplemental methods. Briefly, we extracted total DNA from the swabs, amplified a section of the V4 region of the 16S rRNA gene, and used a MiSeq platform to sequence the fragments [49]. We clustered sequence reads into Amplicon Sequence Variants (ASVs) using the qiime2 platform [50]. ASVs are the most specific lineage designation possible for metabarcoding data. We identified the ASVs taxonomically using an insertion tree method into a GreenGenes2 backbone tree implemented in the “sepp” module in qiime2 [50–54]. We retained singleton ASVs to fully explore the differences in host occupancy across microbial taxa. We used scaling with ranked subsampling implemented in the SRS package in R to address uneven sequencing depths across our samples [55, 56]. All scripts and processed data from this project are available at Zenodo DOI 10.5281/zenodo.16851314.

### Microbial diversity, prevalence, and host specialization across sites

**Figure 1:**
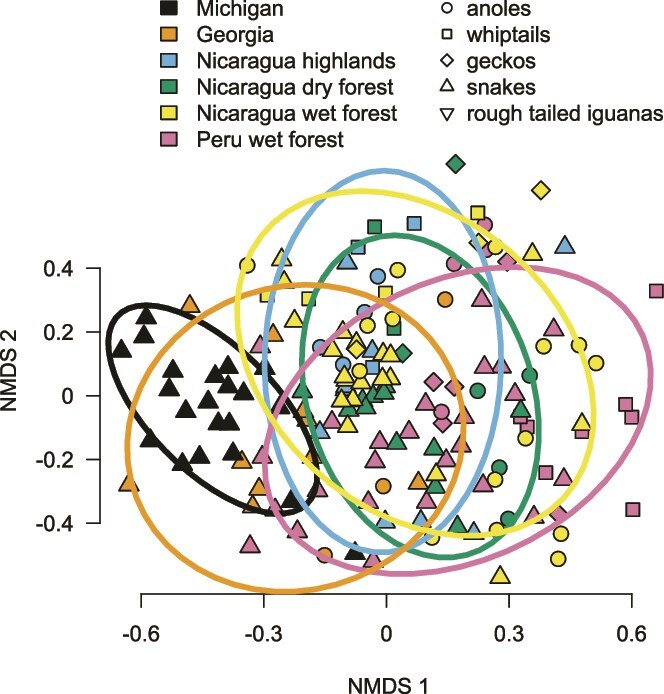
Microbiomes are structured by host family and locality. Pairwise Bray–Curtis distances between the microbiome communities of each host in our sample separate roughly by location, with some clustering by host phylogeny. Ellipses enclose 80% of points per location.

To determine whether our focal bacterial phyla, Proteobacteria and Firmicutes, responded differently to host taxon and geographic separation, we performed a PERMANOVA using the “adonis2” command in the R package vegan 2.6.6.1 [57]. This method uses the Bray-Curtis distance metric to find pairwise distances between the microbial communities of each host, then tests whether communities that share an assigned value of an explanatory variable are closer in pairwise distance space than would be expected by chance [58, 59]. We used sampling site, host family, and a host family-by-site interaction term as explanatory variables. We removed samples from host families with seven or fewer samples, (Fig. 1). We tested all ASVs, then Proteobacteria and Firmicutes ASVs only. We repeated all analyses without the snake-only Michigan samples, as this sampling site confounds host phylogeny and geography. We used the “betadisper” function in vegan 2.6.6.1 to test for homogeneity of variances in the explanatory variables, followed by an ANOVA test for significance [60, 61]. To visualize the relationships, we used the command “metaMDS” implemented in vegan 2.6.6.1 to perform an NMDS analysis on a bacterial community Bray-Curtis distance matrix. We created ellipses encompassing 80% of the points from each location using the function “dataEllipse” from the R package car [62]. To account for multiple comparisons, we used a Bonferroni corrected significance value for all analyses (alpha = 1.31 × 10^−4^).

To understand the relationship between the phylogenetic host generalism and host prevalence of ASVs across sampling sites, we calculated host phylogenetic clustering for each ASV in our subsampled dataset using the “ses.pd” function from the R package picante 1.8.2 [63, 64]. Negative values of this metric represented specialists and positive values represented generalists. The metric is not sensitive to the overall species richness or phylogenetic breadth of the host community. For each ASV, we found mean prevalence and host specialization across all sites in which the ASV occurred.

We hypothesized that high host richness sites would have higher ASV richness and higher host specialization. For each analysis below, we randomly sampled 14 hosts per site (one fewer than our smallest sample size at a site) 100 times, and compared metrics for all ASVs, Proteobacteria ASVs and Firmicutes ASVs to capture differences across bacterial taxa. To test whether ASV richness differed across sites, we compared the median richness of ASVs within hosts and the total ASV richness across all hosts within our six sites. We used median rather than mean because of the large number of singleton ASVs in our dataset. To determine whether patterns of host associations differed across sites, we found the proportion of all ASVs, Firmicutes ASVs, and Proteobacteria ASVs with a host prevalence below five (approximately one third of host individuals) in our randomly sampled datasets. To capture trends in maximum prevalence, we recorded the average number of hosts in which the most prevalent ASV occurred across within-site resamples. Finally, we assessed the relative host phylogenetic specialization of ASVs within each site using the host diversity index from the “ses.pd” function in picante 1.8.2 [63, 64]. We again used the median value for each site for each run. We compared the distributions of maximum prevalence and host specialization of Proteobacteria ASVs to Firmicutes ASVs within each site using a single-sided t-test in base R. For each metric, we ran an ANOVA test between sites using the function “aov” in base R. We then compared pairwise differences between sites using the post-hoc “TukeyHSD” function, also in base R.

### Microbial interactions and host specialization

We expected that generalist, high prevalence ASVs would have weak interactions with other bacterial lineages and host specialist ASVs would have stronger interactions, particularly in high-specialization, high-host richness sites. To test this hypothesis, we identified pairs of ASVs with greater or less than expected overlap in hosts within a site using the R package co-occur 1.3 [65]. The co-occur package used an equation based on combinatorics to calculate the probability that two ASVs both share the observed number of hosts, given each ASV’s prevalence in the dataset [66]. We again subsampled our full dataset down to 14 hosts per site and ran our analyses 100 times on different subsampled datasets. We identified pairs of ASVs that overlapped in hosts either more or less often than would be expected by chance at a 0.01 significance threshold. For each subsampled dataset, we generated a matching null dataset by randomizing the order of entries within each column to decouple interactions between ASVs and hosts while retaining the underlying structure of the community. We used t-tests performed in base R to assess differences between the real and null datasets within sites and between the number of positively and negatively co-occurring pairs within sites. We used an ANOVA followed by post-hoc Tukey tests to assess significant differences in numbers of positive and negative pairs across sites in the real dataset.

However, positively co-occurring pairs might be observed solely due to shared host specificity. To test for this possibility, we used several approaches. First, we compared the prevalence of ASVs in co-occurring pairs between the real and null datasets within sites using t-tests. If host specificity drove positive co-occurrence, we would expect pairs to be made up of low prevalence ASVs in the real dataset. We also tested whether the hosts in which the two ASVs both occurred were more phylogenetically related than would be expected by chance using the “ses.pd” function in picante 1.8.2 [63, 64]. To account for nonrandom patterns of prevalence in our co-occurring ASVs, we generated a second null comparison by randomly sampling ASVs that matched the prevalence of each member of the pair and re-calculating the shared host diversity and ASV patristic distance metrics. If shared host specificity explained co-occurrence, the shared hosts should have been more phylogenetically related in the real compared to null datasets. We again used t-tests to compare the real prevalence values to each of the null datasets, and an ANOVA followed by Tukey tests to compare the real dataset metric values across sites.

To test whether co-occurrence was driven by repeated within-host diversification, we compared the distribution of patristic distances (the distance along branches of a phylogenetic tree between two tips) between co-occurring pairs with the distribution of distances between nearest neighbor ASVs using the function “distTips” in the library adephylo 1.1.16 [67]. If co-occurrence was driven by repeated within-host diversification, co-occurring pairs should have phylogenetic distances similar to the nearest-neighbor values. We also compared the full distribution of patristic distances to the distances between co-occurring pairs. We assessed significance with t-tests within each site.

By inspection, we identified an excess of Proteobacteria-Proteobacteria co-occurring pairs. To test whether this pattern was statistically significant, we found the proportion of all positively co-occurring pairs that were made up of two Proteobacteria ASVs in each run and used t-tests to compare that value to the squared value of the proportion of total Proteobacteria ASVs in each corresponding subsampled dataset, which would be the expected number of Proteobacteria-Proteobacteria pairs that would be drawn at random.

In addition to the pairwise co-occurrence values, we used Newman’s modularity measure to find modules of hosts and ASVs that had two or more hosts within our sites as a method of community detection [68]. Following the same process as for the co-occurrence analysis, we generated a further 50 matched subsampled and null datasets for this analysis. For each, we built a bipartite network using the “metaComputeModules” command in the R package bipartite 2.20 [69–72] in which each ASV is associated with the hosts in which it occurred. ASVs could only be associated with each other through their connections to shared hosts and vice versa. We calculated the modularity of the networks, a statistic that describes the difference between the number of shared host connections between ASVs within modules relative to connections to hosts outside modules. We also identified the number of modules in each run. We compared the values of the real and null datasets within sites using t-tests.

## Results

### Bioinformatics results

Of our 15 943 taxonomically identified ASVs, 3165 were assigned to Proteobacteria at the phylum level, 2534 to Firmicutes (summing ASVs assigned to lower taxonomic groups such as classes or orders formerly classified in the phylum Firmicutes), 1740 to Plancomycetota, and 1312 to Actinobacteria (Fig. S1). At the class level, 2163 ASVs were from Clostridia, 1743 from Alphaproteobacteria, 1419 from Gammaproteobacteria, and 1411 from Plancomycetia.

### Microbial diversity, prevalence, and host specialization across sites

We used PERMANOVA to determine whether sampling site or host family was more predictive of pairwise distances between bacteria communities (Fig. 1). We repeated all PERMANOVAs without the Michigan samples, since Michigan had only snakes, which could lead to a confounding of site and host taxonomy. Overall, we found that pairwise distances between Firmicutes ASVs were best explained by host family, not sampling site. In contrast, site, host family, and their interaction were significant for all ASVs and the Proteobacteria ASVs (see Table S2 for PERMANOVA p-values and R^2^ values). The three predictors were all significant for Firmicutes ASVs when all sites were included, but host family had the highest R^2^ value. Only host family was significant when Michigan was removed (Table S2). Variance was significantly different from equal between groups for sites and for host family for all sites with all ASVs (see Table S2 for p and F values from ANOVA of “betadisper” outputs). The unequal variance could bias our findings. Without Michigan, variance was not significantly different from equal for sites but was for host families (Table S2). For Proteobacteria, no predictor variance was significantly different from equal. Variances were significantly different for all Firmicutes-only analyses (Table S2).

**Figure 2:**
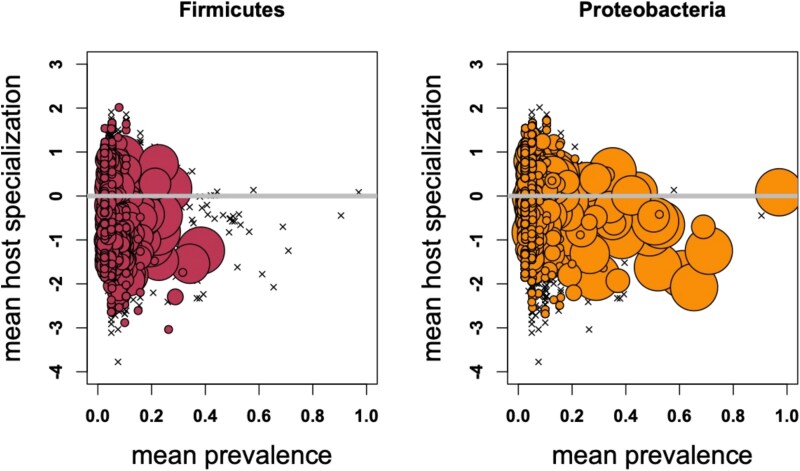
Proteobacteria ASVs reach higher prevalence within communities than Firmicutes ASVs. Points show focal phylum, x’s show all ASVs in the dataset for reference. Point size represents the number of locations in which an ASV occurred, with the largest size representing ASVs that occurred at all six sites. Position on the X axis shows the mean proportion of sampled hosts in which the ASV occurred across the sites in which it was present. Position on the Y axis indicates the mean phylogenetic clustering of hosts at sites where the ASV occurred. Values above zero are host generalists, values below zero are host specialists.

**Figure 3:**
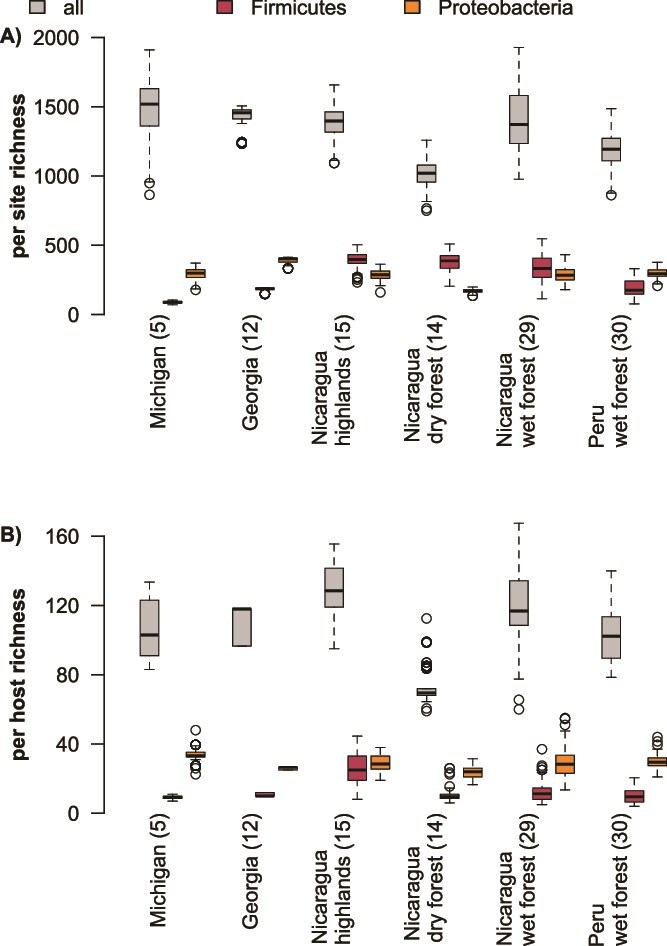
Microbiome species richness from low to high host diversity sampling sites. Site names are on X-axis, numbers in parentheses are the number of sampled host species from the site. While ASV richness does not differ predictably across locations, Firmicutes richness tends to be relatively higher in the tropics across full sites (A) and but not within individual hosts (B).

Based on their distinct ecologies, we hypothesized that ASVs from the phylum Firmicutes would differ in their host associations relative to those from the phylum Proteobacteria. We found that both phyla had phylogenetic host-generalist ASVs but differed in their maximum prevalence per site (Fig. 2). We found significant differences between sites for both within-host and site-wide richness metrics for all ASVs (within host *P* = 5.19 × 10^−103^, site-wide *P* = 1.09 × 10^−87^), Firmicutes ASVs (within host *P* = 3.80x10^−107^, site-wide *P* = 1.30 × 10^−204^), and Proteobacteria ASVs (within host *P* = 3.20 × 10^−51^, site-wide *P* = 5.46 × 10^−191^). We visualized the directionality of these trends (Fig. 3, Table S3) and confirmed the significance of pairwise comparisons using post-hoc Tukey tests (Table S4). We hypothesized that bacterial richness would correlate with host richness. We did not observe a consistent trend in either richness across all hosts in a site (Fig. 3A) or within-host richness (Fig. 3B) for all ASVs or Proteobacteria ASVs. Firmicutes ASVs trended toward relatively higher richness in the tropics at the site level, but not at the within-host level.

**Figure 4:**
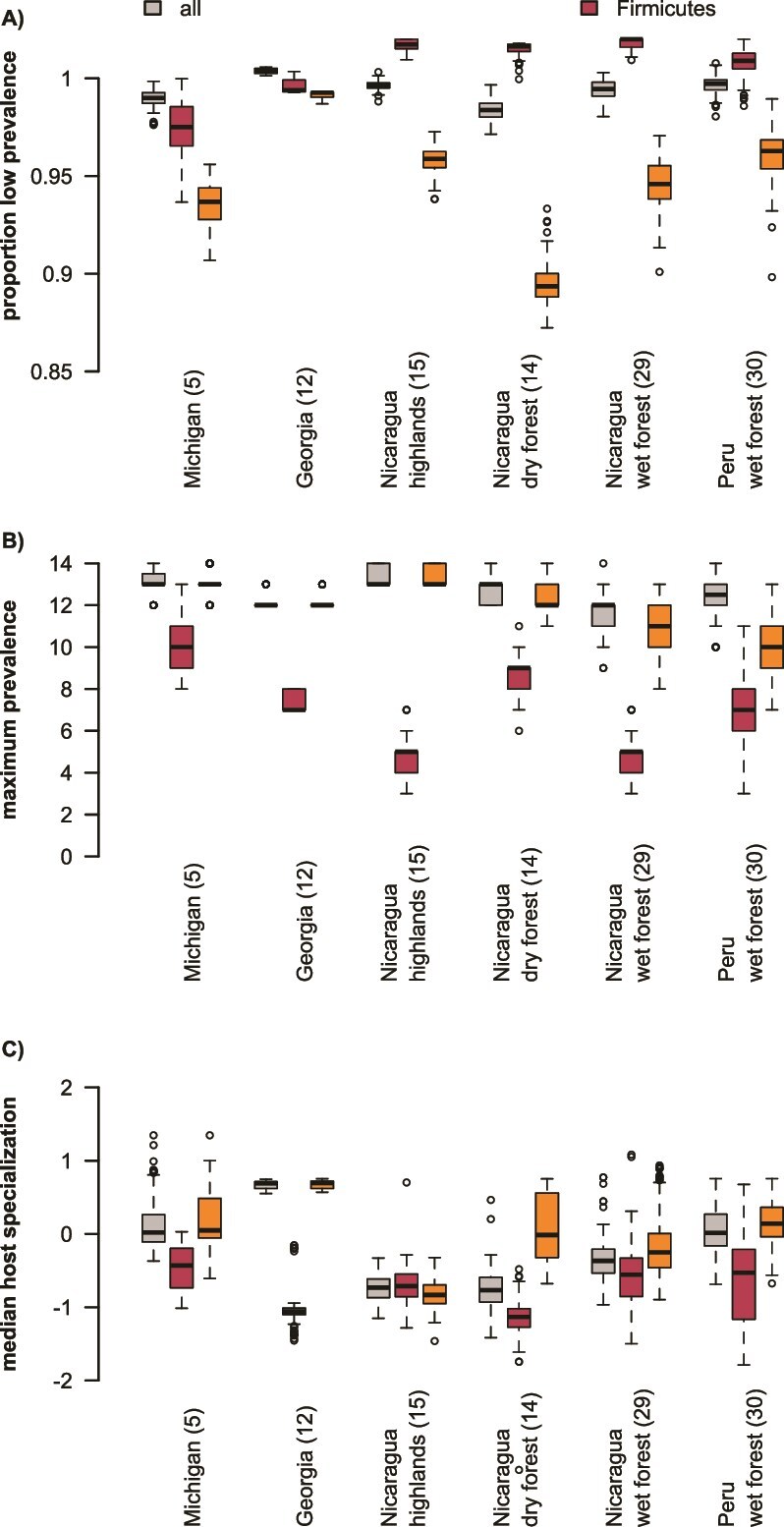
ASV prevalence, but not phylogenetic host specialization, declines in the tropics. Site names are on X-axis, numbers in parentheses are the number of sampled host species from the site. Firmicutes ASVs are uniformly low prevalence (occurring in fewer than five hosts) in tropical locations (A). Maximum prevalence of ASVs (B) declines toward the tropics, and Firmicutes are always less prevalent than Proteobacteria. Median host phylogenetic specialization is location-specific (C), but Firmicutes are more host-specialized than other taxa.

We hypothesized that bacterial lineages would be more host specialized in the tropics. To capture patterns of host prevalence across sampling sites and bacterial phyla, we compared maximum prevalence of ASVs and the proportion of ASVs with a prevalence value below five hosts in a random subsample of 14 hosts. For Firmicutes, tropical sites had a higher proportion of ASVs with prevalence below five hosts (*P* = 5.86 × 10^−228^) while patterns for all ASVs (*P* = 1.94 × 10^−140^) and Proteobacteria ASVs (*P* = 2.24 × 10^−292^) were more site specific (Fig. 4A, Table S3, Table S4). Maximum prevalence of Firmicutes ASVs was lower in the tropics relative to the temperate zone (*P* = 7.14 × 10^−206^), particularly in tropical wet forest sites (Fig. 4B, Table S3, Table S4). Maximum prevalence for Proteobacteria (*P* = 1.86 × 10^−125^) and all ASVs (*P* = 4.05 × 10^−66^) differed in site-specific ways (Fig. 4B, Table S3, Table S4). At all sites, the maximum prevalence of Proteobacteria was higher than the maximum prevalence of Firmicutes (Table S3).

We also compared the phylogenetic similarity of hosts occupied by our ASVs across sites and bacterial phyla. There was no consistent trend across sites for host phylogenetic specialization for any bacterial taxon (Fig. 4C), although between-site differences were statistically significant (Table S3, Table S4, *P* = 8.25 × 10^−193^ for all ASVs, *P* = 5.37 × 10^−33^ for Firmicutes, and *P* = 2.87 × 10^−131^ for Proteobacteria). However, Firmicutes were more phylogenetically restricted than Proteobacteria at five out of our six sites (Table S3). Overall, these results support a higher degree of specialization in host taxonomic breadth and prevalence in Firmicutes than in Proteobacteria.

### Microbial interactions and host specialization

**Figure 5:**
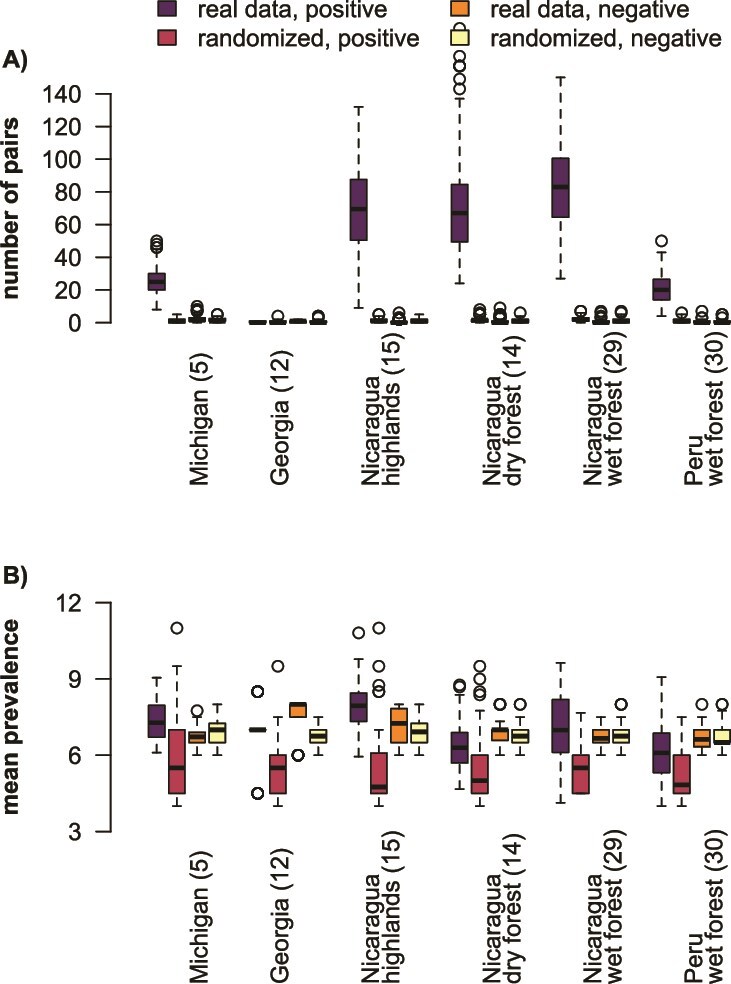
High host diversity communities have more positive interactions between ASVs than would be expected by chance. Pairs of positively co-occurring ASVs are more common in our observed data than the null dataset (A). Negatively co-occurring pairs are similar to their frequency in the null dataset. Locations with higher host diversity have more positively co-occurring pairs. ASVs in positively co-occurring pairs in the real dataset are more prevalent across hosts than those in the null dataset (B). ASVs in negatively co-occurring pairs have similar prevalence to the null dataset.

We hypothesized that generalist bacterial lineages would have weak signatures of interaction with other ASVs, while specialists would have strong interactions. We compared positively and negatively co-occurring pairs of ASVs from 100 random subsamples of 14 hosts per site, each with a paired null dataset. We found significantly more positively co-occurring pairs in the real compared to the null dataset in all sites except Georgia (Table S3 for p-values of within-site pairwise t-tests) and found that the pattern was particularly strong in high host richness sites (ANOVA *P* = 2.57 × 10^−150^, Fig. 5A; Table S3 for ANOVA results; Table S4 for pairwise Tukey test results). The number of negatively co-occurring pairs was also significantly different across sites (ANOVA *P* = 6.97 × 10^−19^, Fig. 5A; Table S4) but did not show a trend with respect to host richness. There were significantly more positively co-occurring pairs than negatively co-occurring pairs across all sites (Table S3). Because the negatively co-occurring pairs were not significantly different from the null dataset values in most sites (Table S3; Fig. 5A), we reported results only for the positively co-occurring pairs in our further analyses.

**Figure 6:**
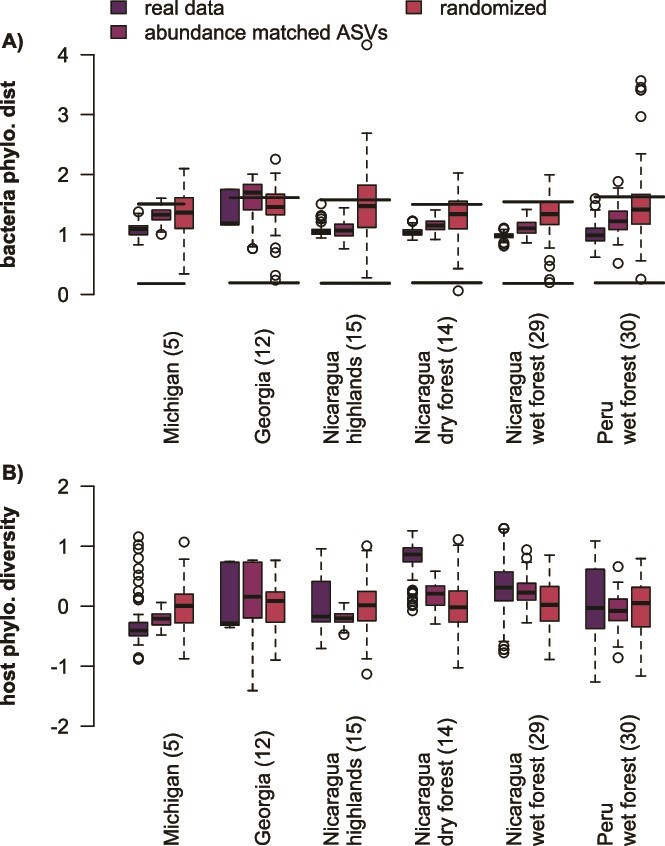
Interacting ASVs are phylogenetically related, but do not cluster in related hosts. (A) Cooccurring pairs are more closely phylogenetically related than would be expected compared to the null dataset. The lower set of horizontal lines indicate mean phylogenetic nearest neighbor distance for ASVs at each site. The upper set of lines represent the mean phylogenetic pairwise distance between ASVs. (B) Phylogenetic diversity of the hosts shared by both ASVs of the co-occurring pairs is not significantly different from the null expectation, indicating that shared host specialization is not driving ASV co-occurrence.

We would expect co-occurring ASVs to have low prevalence and high host specialization indices if their occurrence patterns were driven by shared host specialization. However, the ASVs in positively co-occurring pairs were more prevalent across hosts in the real dataset relative to the null (Fig. 5B; Table S3). We also tested for host phylogenetic clustering using the “ses.pd” distance metric on the hosts shared by both ASVs. The diversity of shared hosts did not differ between real and null datasets or the real and prevalence-matched datasets (Table S3; Fig. 6B). These findings are inconsistent with the idea that shared host specificity drives co-occurrence.

Cooccurring pairs could theoretically arise from repeated within-host diversification, so we compared the phylogenetic distance between co-occurring ASVs. The phylogenetic distances between co-occurring pairs were consistently larger than nearest neighbor distances for each site (Table S3, Fig. 6A), indicating that repeated within-host diversification was not a likely explanation for the pattern. We found that the patristic distance between co-occurring pairs was higher than the nearest neighbor distances but lower than the mean distances (Fig. 6A; Table S3). We found a significant excess of Proteobacteria-Proteobacteria pairs compared to the random expectation in every site (Table S3). This excess of within-phylum pairs reflected our finding that the phylogenetic distance between pairs was smaller than the phylogenetic distance between all pairwise comparisons of ASVs at each site (Table S3, Fig. 6A).

We expanded our analysis of lineage interactions beyond pairs of ASVs using a community detection algorithm. Modularity of our randomized matrices was significantly lower than the modularity of the real matrices at each site (Table S3). Across all sites, our real data had a larger number of modules than the randomized data (Table S3). This finding indicated that the groups of interacting bacterial lineages were larger than the pairwise interactions we detected using the co-occurrence analysis.

## Discussion

We tested three hypotheses about patterns of lineage richness and host specialization in cloacal bacteria across reptile communities from the temperate zone to the tropics. First, we tested whether host taxonomic richness would correlate with bacterial lineage richness. Second, we tested whether bacterial lineages in high host richness communities would be more host-specialized than those in low richness communities. Finally, we tested whether ecological interactions would be strongest between lineages of host-specialized bacteria. We particularly focused on the two most common phyla in our dataset, Firmicutes and Proteobacteria. On average, Firmicutes lineages were more phylogenetically host-specialized and less prevalent across hosts than Proteobacteria lineages. At many sites, one or more Proteobacteria lineages occupied all hosts we sampled. The most prevalent Firmicutes lineages could never achieve this high coverage of host individuals.

Our PERMANOVA analysis showed that the Firmicutes phylum was more structured by host phylogeny while Proteobacteria was equally structured by geographic location and host phylogeny. Generalist bacterial lineages have also been shown to be more geographically structured than specialists in studies of free-living microbial communities [46, 73–75]. One possible explanation for this outcome could arise if specialist lineages are vertically inherited, while generalist lineages are acquired from the environment. Such a process could explain the patterns we observed if local environmental conditions filter the environmentally acquired bacterial lineages more stringently between sites than do the hosts supporting the vertically inherited bacterial lineages. More large-scale sampling of host-associated and free-living microbes will be necessary to identify the mechanisms behind the patterns we observed.

In our first hypothesis, we asked whether bacteria lineage richness correlated with host species richness. In general, bacterial lineages did not increase in richness along with their hosts. However, lineages in the phylum Firmicutes were higher in richness in most of our tropical sites, similar to patterns found in pathogens and parasite taxa [44, 45]. While we found a difference in ASV richness between temperate and tropical sites, we did not see a linear increase in diversity with our host community richness, indicating that temperate-tropical richness differences may be driven by mechanisms we do not test here. Future work could explore nonlinear relationships between host and bacterial community diversity. Our data show patterns that directly contrast with free-living bacteria assemblages, in which generalist microbes, rather than specialist, are more diverse near the equator [46]. This contrast may point to a fundamental difference in host filtering compared to the ecological pressures experienced by free-living bacteria communities. Future work will be necessary to determine whether the patterns we find are specific to the cloacal microbiome or are common in microbial communities associated with other tissue types.

Second, we tested whether bacterial lineages were more host specialized when host richness was high. We did not detect a consistent trend in host phylogenetic specialization across sites but did find that Firmicutes were consistently more phylogenetically specialized than Proteobacteria. In addition to host phylogeny, we compared prevalence across host individuals for all ASVs. We found that bacterial lineages from all taxa had lower maximum prevalence in tropical sites, and Firmicutes had a higher proportion of low-prevalence lineages in the tropics.

This finding coupled with the increase in Firmicutes lineage richness at the site level in the tropics may indicate Firmicutes and Proteobacteria respond differently to high-diversity host communities. Previous work has found a trend toward higher investment in immune defenses by vertebrates toward the equator [76]. If Firmicutes lineages on average respond more strongly to host immune investment than Proteobacteria lineages, then increases in host immune investment in combination with higher host diversity could explain our observed trends. Our pattern of lower maximum prevalence across all ASVs could be consistent with an absolute upper limit on phylogenetic host breadth. However, all sites except our most northerly sampling area had similar maximum phylogenetic breadth between hosts. We therefore hypothesize that specialists could be more competitively dominant within some hosts compared to generalists in high host richness tropical sites, reducing the overall maximum prevalence of generalists in these communities. Similar competition-driven processes have been observed in plant-microbe systems [77], and are frequently observed in multicellular species assemblages [78–81]. Much future work will be necessary to rigorously test this hypothesis, which cannot be directly tested by our dataset [82, 83].

Finally, we tested whether host specialist bacterial lineages were more likely to show signatures of ecological interactions with other lineages across host communities. We hypothesized that groups of specialist lineages would be brought into contact frequently in their shared hosts, leading to coevolutionary interactions. In contrast to our hypothesis, we found that the strongest signatures of interaction were among lineages that were some of the most prevalent in their communities and were disproportionately from the phylum Proteobacteria. The relatively lower incidence of significantly co-occurring Firmicutes-Firmicutes pairs could point to a tradeoff between maintaining host specificity and participating in mutualistic interactions with other bacteria taxa. In addition, scores of community modularity, a measure of how tightly groups of lineages interact, increased in the tropics in our dataset. This result is counterintuitive given the higher host species richness of tropical sites but could be consistent with our observation that low-prevalence ASVs are more numerically dominant in the tropics.

Biological explanations for the pattern of co-occurring lineages could include co-operative processes like biofilm formation [84] or shared metabolites [85, 86], both of which could buffer bacteria against host immune defenses or competition from specialists [87, 88]. Positive associations between bacterial lineages are widespread in the vertebrate gut microbiome, including in mice [84], fish [85], and humans [86, 88]. Additionally, the outcome could reflect shared food resources, if hosts have similar diets [89–91]. However, if shared dietary specializations explained bacterial lineage co-occurrence, we would be more likely to see co-occurrence among more specialist bacterial lineages, not the most generalist lineages. Another potential explanation is shared acquisition of generalist environmental bacteria. Our Georgia site had the most geographically distributed sampling. It also had fewer positive and more negatively co-occurring pairs than other datasets, indicating a role for spatial effects in our bacteria interaction findings. However, we did not have the statistical power to test this hypothesis with our dataset. Future work across a greater variety of distance lags will be necessary to determine the impacts of local processes on bacteria lineage interactions in the reptile cloacal microbiome.

### Conclusions and future directions

We demonstrate that even the most generalist host-associated bacteria have lower host prevalence in extremely high richness host communities. These limitations could be imposed by host immune responses, nutrient availability, or competition from other members of the microbial community [27–30, 92]. We hypothesize that a combination of these factors drives our observed patterns, in which microbes that are maladapted to a host environment are at a competitive disadvantage and can be excluded by better-adapted specialists. One possible mechanism could come from the observation that bacteria are biased towards genome size reduction as they specialize [93, 94], while larger genomes facilitate habitat generalism and resilience to environmental perturbations. When this process is adaptive, it is referred to as genome streamlining [95, 96]. Genome streamlining in bacterial lineages within interconnected microbial networks was first observed in marine Proteobacteria [95, 96]. We hypothesize that tropical environments or hosts might favor genome streamlining among interacting microbial networks more than temperate host communities.

We found that the preponderance of interactions, particularly in high host richness sites, were between members of the phylum Proteobacteria (Fig. 7). This result stands in contrast to other studies of free-living microbial communities that find an overall pattern of co-occurring ASVs being habitat specialists [97]. In our dataset, the interacting pairs were relatively distantly related within their phylum. We hypothesize that this pattern hints at a “Goldilocks zone” of evolutionary divergence for mutualistic interactions. Closely related pairs might be more likely to compete than co-operate, while distantly related pairs may not share enough metabolic pathways in common to successfully co-facilitate while not directly competing. This outcome is in contradiction to our hypothesis that interactions would be stronger between host specialist microbes. Instead, our observed patterns may reflect a tradeoff between specializing on host interactions (used by Firmicutes) compared to investing in interactions with other bacteria (used by Proteobacteria).

## Supplementary Material

ISME_supp_figures_sub3_ycaf146

TableS1_May_ycaf146

TableS2_May_ycaf146

TableS3_May_ycaf146

TableS4_May_ycaf146

TableS5_May_ycaf146

LG_supplemental_methods_sub3_ycaf146

## Data Availability

All custom scripts and processed data from this project are available at Zenodo DOI 10.5281/zenodo.16851314. Raw fastq files are available on NCBI’s Short Read Archive under BioProject PRJNA1265172.
