# Supplementary material for: Increased host diversity limits bacterial generalism but may promote microbe-microbe interactions
Source: ISME Commun. 2025 Aug 23;5(1):ycaf146. doi: 10.1093/ismeco/ycaf146 (PMC12448418; doi:10.1093/ismeco/ycaf146)
Supplement: ISME_supp_figures_sub3_ycaf146 [file isme_supp_figures_sub3_ycaf146.docx]

**Figure S1**: proportions of the reads assigned to the four m
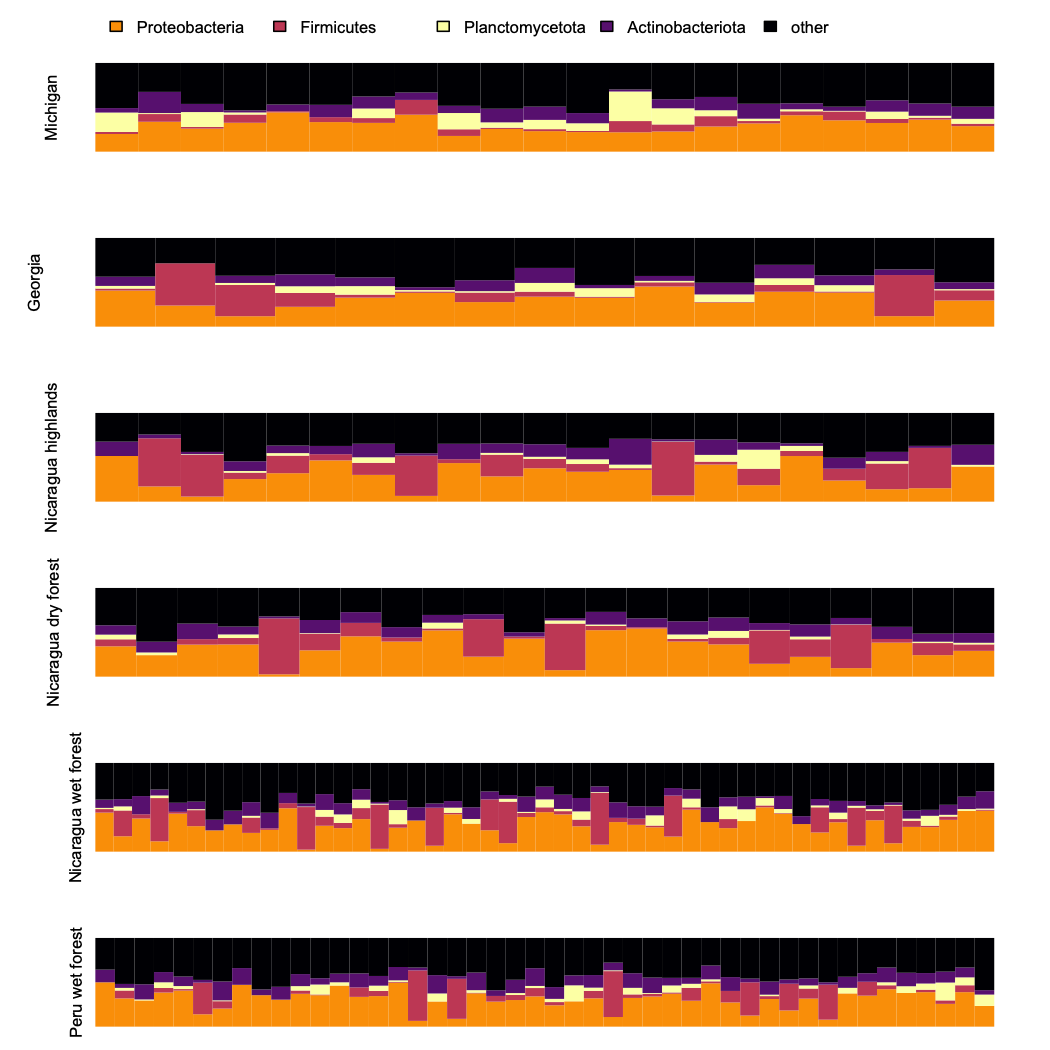
ost common phyla relative to other phyla across the dataset.
