# Supplementary material for: Increased host diversity limits bacterial generalism but may promote microbe-microbe interactions
Source: ISME Commun. 2025 Aug 23;5(1):ycaf146. doi: 10.1093/ismeco/ycaf146 (PMC12448418; doi:10.1093/ismeco/ycaf146)
Supplement: LG_supplemental_methods_sub3_ycaf146 [file lg_supplemental_methods_sub3_ycaf146.docx]

*Host species richness methods*

To quantify the richness of all potential host species and higher taxa at our sampling sites, we used the IUCN ‘scaled reptile’ range polygons to find the number of ranges that intersected the mean latitude and longitude of the capture locations at each of our sampling sites (Table 1) [1].

*Sampling methods*

We captured animals using hand grabs for lizards and snakes, funnel and pitfall trapping, cover boards, and lassoing with surgical silk for lizards [2]. We used opportunistic foot surveys during day and night at all sites, funnel and pitfall trapping in Nicaragua and Peru, and cover boards in Michigan. Funnel trapping, pitfall trapping, and cover boards can all be helpful for sampling leaf litter and fossorial species. Our lack of a trapping method in Georgia may have altered the composition of our sampled community for that site relative to others.

Where possible, we report the exact GPS coordinates of the captured hosts. In Table 1, we report the mean of all latitudes and mean of all longitudes and the maximum spread of GPS points for each site. We identified hosts to species in the field. We took cloacal swabs to sample the microbiota [3]. Swabs were stored in RNAlater in the field and kept at ambient temperature away from direct sun for 1-5 weeks. Samples were then stored at -20 °C until DNA extraction. We took voucher specimens for our Nicaraguan and Peruvian samples, so species identification was confirmed at time of accession. All Nicaraguan voucher specimens were deposited at the University of Michigan Museum of Zoology (accession numbers available in Table S1). Half of the Peruvian voucher specimens were deposited in the Museo de San Marcos in Lima, Peru and half at the University of Michigan Museum of Zoology.

To quantify host phylogeny, we used a phylogenetic tree from Tonini et al. 2016 [4]. Some species in our dataset were not represented on the tree. For these species, we chose a congeneric match that was represented in the tree (see Supplemental Table 5 for species IDs and chosen matches). All of our species had a closer phylogenetic relative in the tree than their closest phylogenetic relative in our dataset, so this approach did not bias our methods that use host phylogenetic pairwise distance.

Because tropical seasonality differs from temperate seasons, we cannot directly compare sampling times. In the temperate zone, we sampled in spring, as our host species were actively foraging following the winter hibernation (Table S1). In the tropics, we sampled at the start of the wet season (November in Peru, May in Nicaragua). Tropical reptiles do not all hibernate during the dry season, but many species dramatically increase activity and feeding at the start of the wet season, leading to a set of behaviors that share similarities to those displayed by temperate species in spring [5, 6].

*Laboratory methods*

To isolate cells from swabs, we incubated them at 65 °C for 12 hours with Proteinase K and buffer ATL in a rocking incubator, including several rounds of vortexing. Next, we extracted total DNA from the cell suspension using Qiagen DNEasy blood and tissue kits (Product # 69506). DNEasy kits are not targeted toward extracting bacterial DNA, and in particular lack the bead beating step that is used to break down the tough cell walls of gram positive bacteria [7]. While this is a concern for our data, we chose to maintain a consistent extraction protocol throughout our study to preserve our ability to compare across our sampled sites.

We sequenced the samples on a MiSeq platform (San Diego, CA) with version 2 reagents at the University of Michigan Microbiome Core Facility, using a primer set developed to amplify 252 bp from the 16S rRNA V4 region (primer pair 16SV4f/16SV4r) [8]. Each sequencing run had a positive (mock community) and negative (water) control provided by the sequencing core [9].

*Sampling and laboratory methods caveats*

Our study has important methodological caveats. First, we sampled locations only in one season. Patterns of host-bacteria and bacteria-bacteria interactions might change profoundly between seasons, but we would not be able to detect such a change. Second, extraction methods can impact the taxa recovered from cloacal swabs [10], meaning that our sequenced samples are likely not a complete picture of the cloacal microbiome. We used an extraction method that did not have a bead beating step, which is used to break apart the harder cell walls of some bacteria [7]. Therefore, our sequencing results could be biased against those bacteria.

In addition, the physical size of our sampled areas was not equal, and our coverage within areas was constrained by trail availability. These factors could impact our conclusions. Third, our tropical samples were held for different lengths of time in ambient temperatures in the field. Lengths of time at ambient temperature could vary between three days and four weeks. This could have impacted some of our within-site analyses, or the tropical-temperate comparisons of lineage richness by reducing our richness estimates for our tropical locations. The larger geographic spread for our Georgia samples (Table 1) could have impacted the positive and negative interactions we detected between ASV lineages. Specifically, small-scale geographic structuring of lineages might have led to a higher rate of negative cooccurrences between ASVs in this sampling location.

*Bioinformatics*

We classified our ASVs using the ‘sepp’ command implemented in qiime2 using the GreenGenes2 backbone reference taxonomy [11], specifically the ‘2022.10.backbone.sepp-reference.qza’ object available from the GreenGenes2 ftp release (https://ftp.microbio.me/greengenes_release/2022.10-rc1/). GreenGenes2 is an expansion of the initial GreenGenes database and includes both genomic and full length 16S rRNA sequences. GreenGenes2 is a larger reference database than SILVA or GDTB. The GreenGenes2 backbone phylogenetic tree is derived from the Web of Life, a supertree constructed from over 300 genes. By using the supertree reference phylogeny, GreenGenes2 avoids the biases that can arise from building a reference tree from one single gene or genomic region [12].

In qiime2, we used a truncation length of 150 bp, pooled our ASVs independently, and identified chimeras using the ‘pooled’ method. All qiime2 commands are included in the R script that includes our data processing code. We processed the qiime2 sepp tree output and dada2 output in R using the R package qiime2R, and used base R to remove non-bacteria sequences from our reference tree, clip out reference sequence tips, and remove non-bacteria ASVs from our host x ASV tree derived from dada2 [14, 15]. We then found the taxonomic identification of our ASVs by determining whether they were downstream from a taxonomy label annotated on a node of the reference tree. The phylum Firmicutes (also referred to as Bacillota) is not annotated on the reference tree. To capture ASVs that should be assigned to Firmicutes, we found all lower-order taxa annotated on our tree that have in the past been assigned to Firmicutes. We used the NCBI taxonomy database and the Lawrence Berkeley Labs fast.genomics database (fast.genomics.lbl.gov) to check the lower-order taxa. We assigned ASVs belonging to the class Clostridia, the orders Acidaminococcale, Bacillales, Christensenellales, Culicoidibacterales, DSM-22653, Erysipelotrichales, Eubacteriales, Lactobacillales, Oscillospirales, Peptostreptococcales, Selenomonadales, and Sporomusales, the families Amphibacillaceae, Paenibacillaceae, Turicibacteraceae, Veillonellaceae, and the genera Coprosoma, Faecimonas, Scybalousia, and Tumebacillus.

After qiime2 processing, we used the host by ASV matrix, including negative controls, generated by the dada2 module to identify potential contaminants using the R package decontam [13]. Negative controls were water added to the sample plate prior to lane prep. We were not able to include negative controls for our sample buffer or extraction process, which could impact the validity of our findings. We identified potential contaminants from the lane prep and sequencing process using both frequency and prevalence approaches, and removed all ASVs identified by either algorithm from our dataset for future analyses.

To determine whether we needed to process our data to account for differing read depths, we plotted ASV accumulation curves for each host (Figure Supp Methods 1) and plotted the number of ASVs compared to the number of sequencing reads for each host (Figure Supp Methods 2). We used the SRS package in R [16, 17] to perform scaling with ranked subsampling on our data, with a cutoff value of 25000 reads per host. We chose this value as a compromise between coverage depth and the number of hosts we retained in our dataset (Figure Supp Methods 3). We removed any hosts with lower read counts than our cutoff and adjusted our host by ASV matrix counts based on the results of the SRS analysis. We used the resulting matrix throughout our analyses and include it with our code for reproducibility at Zenodo DOI 10.5281/zenodo.16851314.

**Results**

Of the 15,721 ASVs identified by dada2, 116 aligned to Archaea references. With these removed, we retained 15,605 bacteria ASVs. The decontam package identified 31 ASVs as potential contaminants using the frequency approach and 13 from the prevalence approach in the decontam package. None of the ASVs overlapped, so we removed all 44 from our dataset, leaving us with 15,561 ASVs. Our sampled hosts ranged from read depths of 59,206 to 2,459 reads. We removed the 31 hosts with fewer than 25000 reads, for a total of 215 hosts remaining in our final dataset.

**Works Cited**

1. IUCN. IUCN Scaled Reptile digital distribution maps. 2022. https://www.iucnredlist.org/resources/spatial-data-download: IUCN spatial data, 2022.

2. Lettink M, Hare KM. Sampling techniques for New Zealand lizards. New Zealand Lizards. Springer, 2016, 269–291.

3. Colston TJ, Noonan BP, Jackson CR. Phylogenetic analysis of bacterial communities in different regions of the gastrointestinal tract of *Agkistrodon piscivorus*, the cottonmouth snake. *PLOS ONE* 2015;**10**:e0128793. https://doi.org/10.1371/journal.pone.0128793

4. Tonini JFR et al. Fully-sampled phylogenies of squamates reveal evolutionary patterns in threat status. *Biological Conservation* 2016;**204**:23–31. https://doi.org/10.1016/j.biocon.2016.03.039

5. Brown GP, Shine R. Influence of weather conditions on activity of tropical snakes. *Austral Ecology* 2002;**27**:596–605. https://doi.org/10.1046/j.1442-9993.2002.01218.x

6. Rahman SC et al. Monsoon does matter: annual activity patterns in a snake assemblage from Bangladesh. *The Herpetological Journal* 2013;**23**:203–208.

7. De Boer R et al. Improved detection of microbial DNA after bead-beating before DNA isolation. *Journal of Microbiological Methods* 2010;**80**:209–211. https://doi.org/10.1016/j.mimet.2009.11.009

8. Kozich JJ et al. Development of a dual-Index sequencing strategy and curation pipeline for analyzing amplicon sequence data on the MiSeq Illumina sequencing platform. *Applied and Environmental Microbiology* 2013;**79**:5112–5120. https://doi.org/10.1128/AEM.01043-13

9. Kozich J et al. 16S rRNA Sequencing with the Illumina MiSeq: library Generation, QC, & xequencing. 2013. 2013.

10. Hoffbeck C et al. 16S rRNA gene‐based meta‐analysis of the reptile gut microbiota reveals environmental effects, host influences and a limited core microbiota. *Molecular Ecology* 2023;**32**:6044–6058. https://doi.org/10.1111/mec.17153

11. McDonald D et al. Greengenes2 unifies microbial data in a single reference tree. *Nat Biotechnol* 2024;**42**:715–718. https://doi.org/10.1038/s41587-023-01845-1

12. Rajendhran J, Gunasekaran P. Microbial phylogeny and diversity: Small subunit ribosomal RNA sequence analysis and beyond. *Microbiological Research* 2011;**166**:99–110. https://doi.org/10.1016/j.micres.2010.02.003

13. Davis N et al. Simple statistical identification and removal of contaminant sequences in marker-gene and metagenomics data. *bioRxiv* 2017;221499. https://doi.org/10.1101/221499

14. Bisanz JE. qiime2R: Importing QIIME2 artifacts and associated data into R sessions. 2018. 2018.

15. R Core Team. R: A language and environment for statistical computing. 2021. Vienna, Austria: R Foundation for Statistical Computing, 2021.

16. Beule L, Karlovsky P. Improved normalization of species count data in ecology by scaling with ranked subsampling (SRS): application to microbial communities. *PeerJ* 2020;**8**:e9593. https://doi.org/10.7717/peerj.9593

17. Heidrich V, Karlovsky P, Beule L. ‘SRS’ R Package and ‘q2-srs’ QIIME 2 Plugin: Normalization of Microbiome Data Using Scaling with Ranked Subsampling (SRS). *Applied Sciences* 2021;**11**:11473. https://doi.org/10.3390/app112311473

Supplemental Methods Figures

**
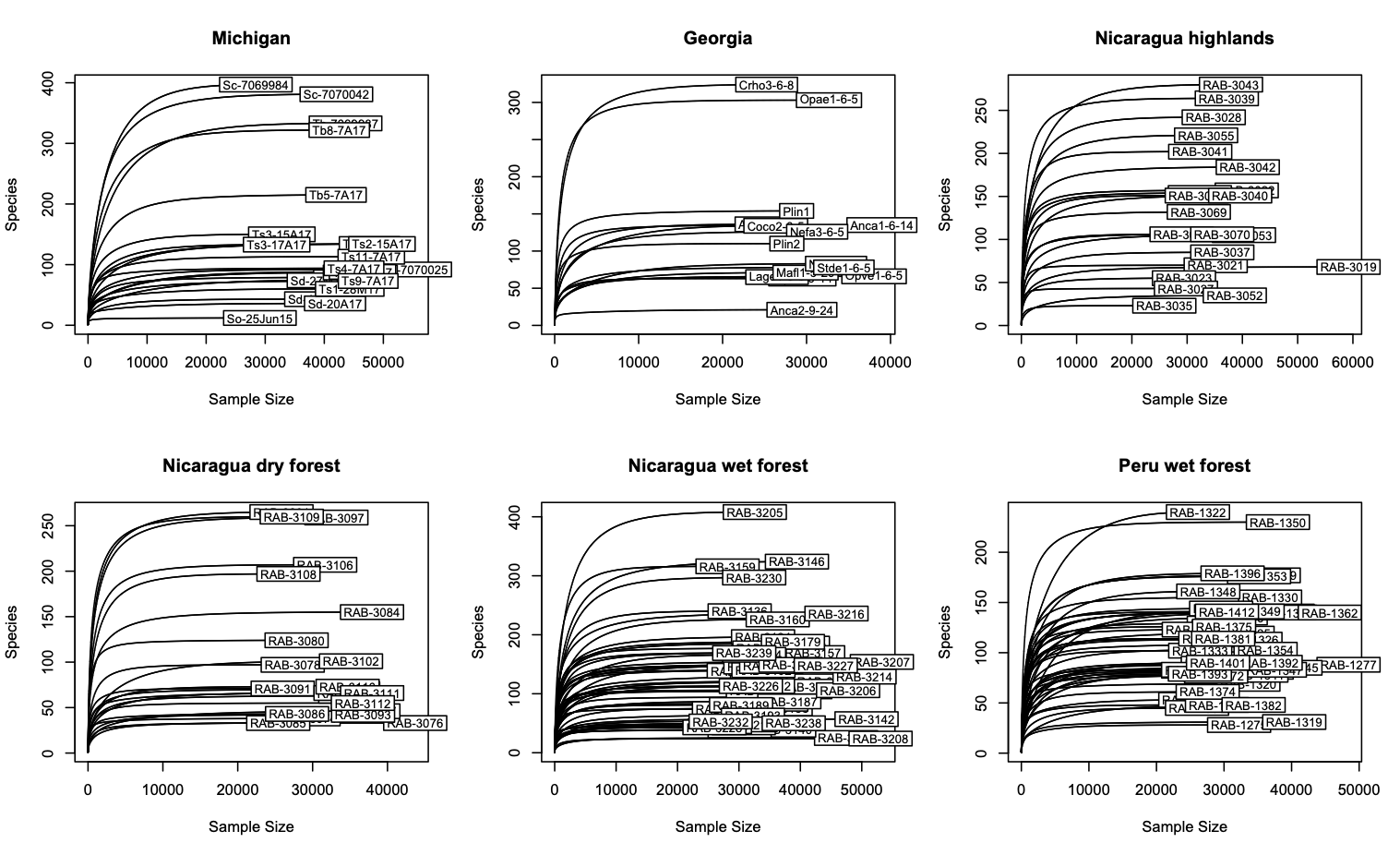
**

**Figure Supp Methods 1:** ASV accumulation curves across all sampling sites. Curves reach maximum ASV diversity by ~20,000 reads.

**
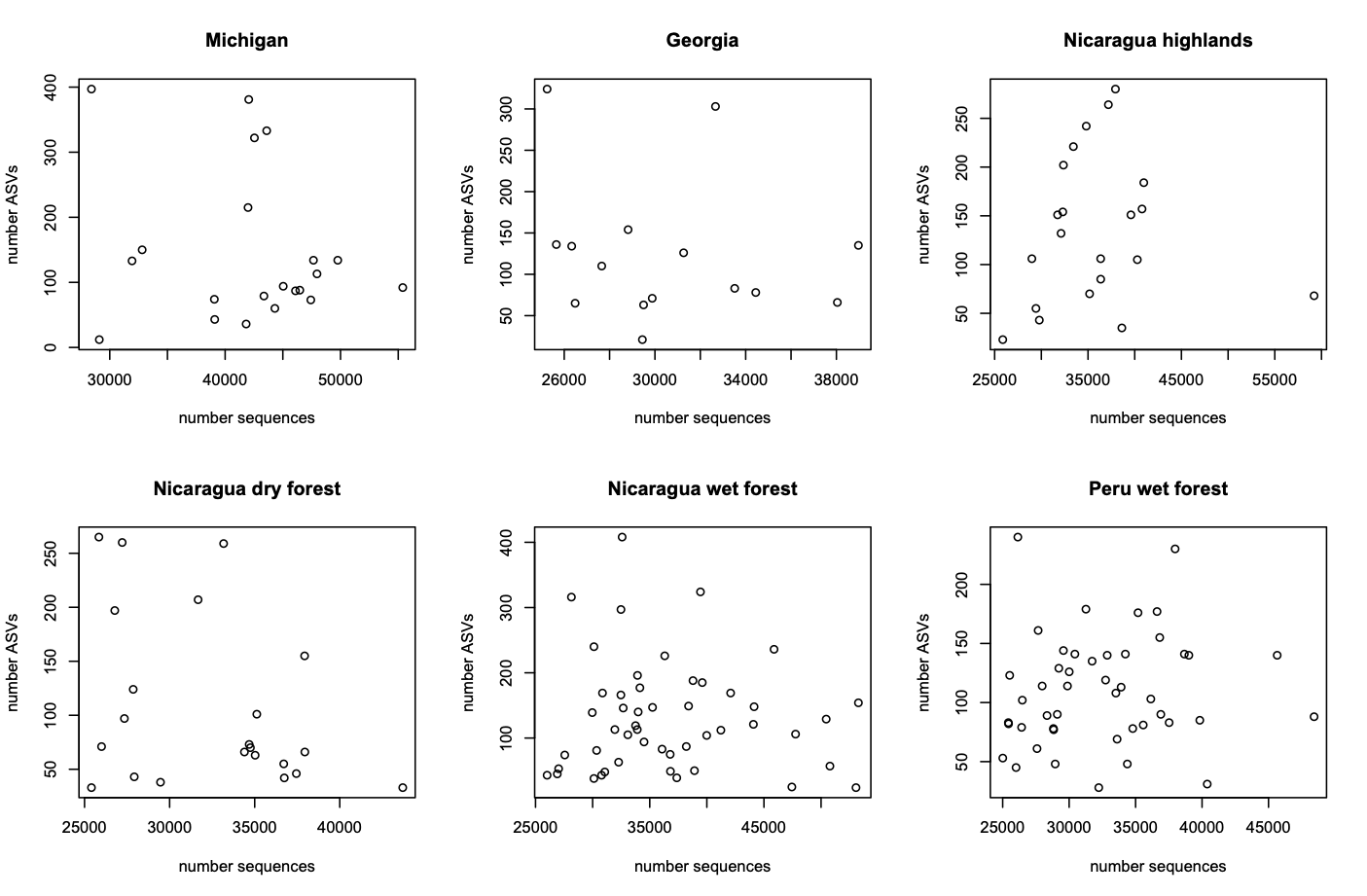
**

**Figure Supp Methods 2:** there is no strong relationship between numbers of ASVs and numbers of reads.


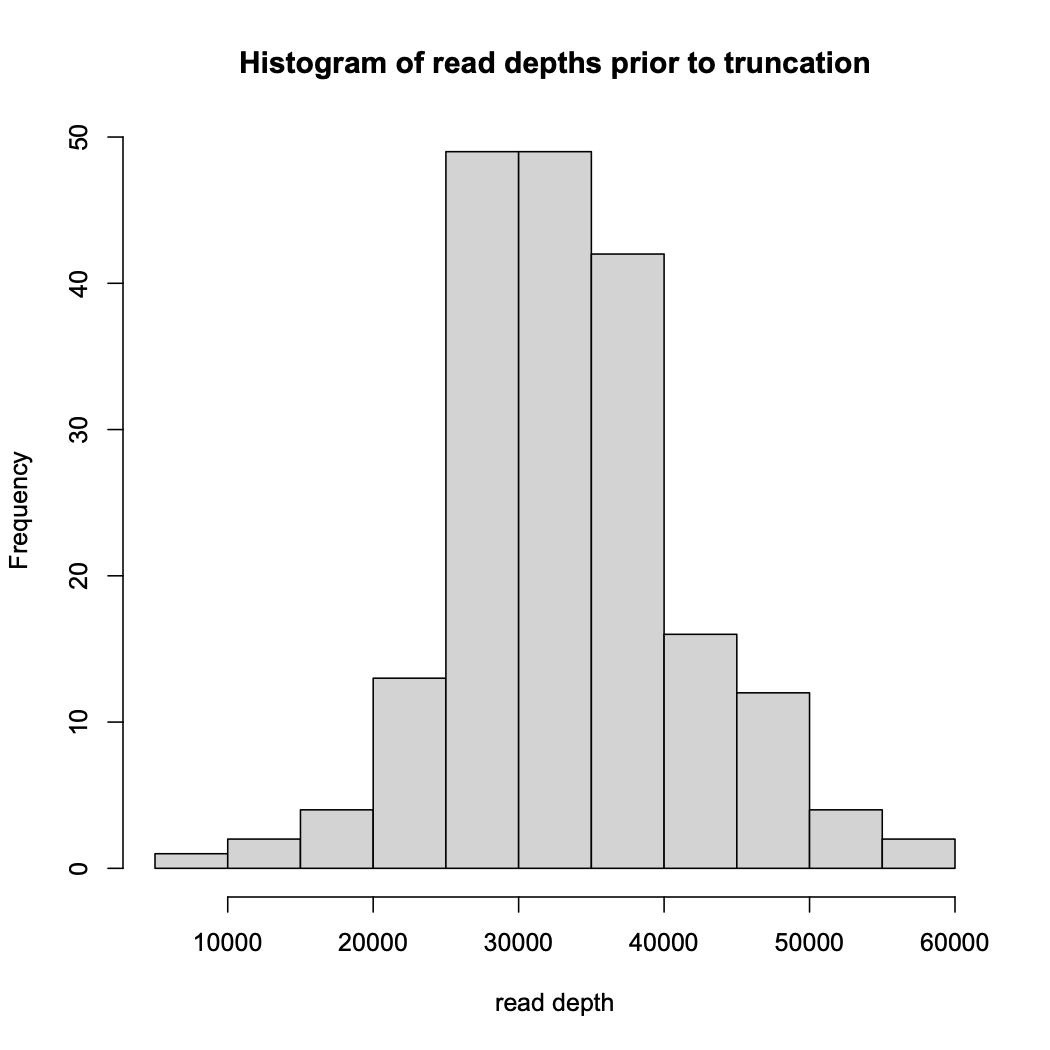


**Figure Supp Methods 3**: histogram of numbers of reads per host across the dataset.

**Supplemental table 5: relationship between sampled species and species used from Tonini tree. CSV available in Zenodo repository**

| **my species** | **tonini** |  |  |
| --- | --- | --- | --- |
| **Alopoglossus_angulatus** | Alopoglossus_angulatus |  |  |
| **Ameiva_ameiva** | Ameiva_ameiva |  |  |
| **Ameiva_festiva** | Holcosus_festivus |  |  |
| **Ameiva_undulata** | Holcosus_undulatus |  |  |
| **Anilius_scytale** | Anilius_scytale |  |  |
| **Anolis_biporcatus** | Anolis_biporcatus |  |  |
| **Anolis_capito** | Anolis_capito |  |  |
| **Anolis_carolinensis** | Anolis_carolinensis |  |  |
| **Anolis_cupreus** | Anolis_cupreus |  |  |
| **Anolis_dariensis** | Anolis_cupreus |  |  |
| **Anolis_fuscoauratus** | Anolis_fuscoauratus |  |  |
| **Anolis_lemurinus** | Anolis_lemurinus |  |  |
| **Anolis_limifrons** | Anolis_limifrons |  |  |
| **Anolis_nitens** | Anolis_chrysolepis |  |  |
| **Anolis_ortonii** | Anolis_ortonii |  |  |
| **Anolis_oxylophus** | Anolis_oxylophus |  |  |
| **Anolis_punctatus** | Anolis_punctatus |  |  |
| **Anolis_quaggulus** | Anolis_quaggulus |  |  |
| **Anolis_sagrei** | Anolis_sagrei |  |  |
| **Anolis_sericeus** | Anolis_sericeus |  |  |
| **Anolis_sp1** | Anolis_limifrons |  |  |
| **Anolis_tropidonotus** | Anolis_tropidonotus |  |  |
| **Aspidoscelis_deppii** | Aspidoscelis_deppei |  |  |
| **Aspidoscelis_motaguae** | Aspidoscelis_communis |  | Barley et al. 2019 |
| **Atractus_elaps** | Atractus_elaps |  |  |
| **Atractus_flammigerus** | Atractus_flammigerus |  |  |
| **Atractus_sp** | Atractus_flammigerus |  |  |
| **Atropoides_indomitus** | Atropoides_indomitus |  |  |
| **Bachia_dorbignyi** | Bachia_dorbignyi |  |  |
| **Bachia_trisanale** | Bachia_trisanale |  |  |
| **Basiliscus_plumifrons** | Basiliscus_plumifrons |  |  |
| **Basiliscus_vittatus** | Basiliscus_vittatus |  |  |
| **Bothriechis_schlegelii** | Bothriechis_schlegelii |  |  |
| **Bothriopsis_bilineata** | Bothrops_taeniata |  | Bernarde et al. 2021 |
| **Bothrops_asper** | Bothrops_asper |  |  |
| **Bothrops_brazili** | Bothrops_brazili |  |  |
| **Cercosaura_argulus** | Cercosaura_argulus |  |  |
| **Cercosaura_eigenmanni** | Cercosaura_eigenmanni |  |  |
| **Chironius_fuscus** | Chironius_fuscus |  |  |
| **Chironius_grandisquamis** | Chironius_grandisquamis |  |  |
| **Chironius_multiventris** | Chironius_multiventris |  |  |
| **Chironius_scurrulus** | Chironius_scurrulus |  |  |
| **Clelia_clelia** | Clelia_clelia |  |  |
| **Coleonyx_mitratus** | Coleonyx_mitratus |  |  |
| **Coluber_constrictor** | Coluber_constrictor |  |  |
| **Conophis_lineatus** | Conophis_lineatus |  |  |
| **Corallus_hortulanus** | Corallus_hortulanus |  |  |
| **Corytophanes_cristatus** | Corytophanes_cristatus |  |  |
| **Crotalus_adamanteus** | Crotalus_adamanteus |  |  |
| **Crotalus_horridus** | Crotalus_horridus |  |  |
| **Dendrophidion_dendrophis** | Dendrophidion_dendrophis |  |  |
| **Dendrophidion_percarinatum** | Dendrophidion_percarinatum |  |  |
| **Diadophis_punctatus** | Diadophis_punctatus |  |  |
| **Dipsas_articulata** | Dipsas_articulata |  |  |
| **Dipsas_catesbyi** | Dipsas_catesbyi |  |  |
| **Drepanoides_anomalus** | Drepanoides_anomalus |  |  |
| **Drymarchon_melanurus** | Drymarchon_corais |  |  |
| **Drymobius_rhombifer** | Drymobius_rhombifer |  |  |
| **Drymoluber_dichrous** | Drymoluber_dichrous |  |  |
| **Enulius_flavitorques** | Xenodon_merremi |  | Zaher et al. 2009, Myers 2014 |
| **Enulius_sclateri** | Xenodon_merremi |  |  |
| **Epicrates_cenchria** | Epicrates_cenchria |  |  |
| **Erythrolamprus_sp** | Erythrolamprus_reginae |  | co-occurs |
| **Gonatodes_albogularis** | Gonatodes_albogularis |  |  |
| **Gonatodes_hasemani** | Gonatodes_hasemani |  |  |
| **Gonatodes_humeralis** | Gonatodes_humeralis |  |  |
| **Gymnopthalmus_speciosus** | Gymnophthalmus_speciosus |  |  |
| **Helicops_angulatus** | Helicops_angulatus |  |  |
| **Heterodon_nasicus** | Heterodon_nasicus |  |  |
| **Imantodes_cenchoa** | Imantodes_cenchoa |  |  |
| **Imantodes_chenchoa** | Imantodes_cenchoa |  |  |
| **Imantodes_lentiferus** | Imantodes_lentiferus |  |  |
| **Iphisa_elegans** | Iphisa_elegans |  |  |
| **Kentropyx_pelviceps** | Kentropyx_pelviceps |  |  |
| **Lampropeltis_abnorma** | Lampropeltis_abnorma |  |  |
| **Lampropeltis_getula** | Lampropeltis_getula |  |  |
| **Lepidophyma_flavimaculatum** | Lepidophyma_flavimaculatum |  |  |
| **Leptodeira_annulata** | Leptodeira_annulata |  |  |
| **Leptodeira_nigrofasciata** | Leptodeira_nigrofasciata |  |  |
| **Leptodeira_rhombifera** | Leptodeira_annulata |  | Barrio-Amoros et al. 2019 |
| **Leptodeira_septentrionalis** | Leptodeira_septentrionalis |  |  |
| **Leptodrymus_pulcherrimus** | Leptodrymus_pulcherrimus |  |  |
| **Leptophis _ahaetulla** | Leptophis_ahaetulla |  |  |
| **Masticophis_flagellum** | Coluber_flagellum |  |  |
| **Mastigodryas_alternatus** | Mastigodryas_melanolomus |  | Montingelli et al. 2019 |
| **Mesaspis_moreletti** | Mesaspis_gadovii |  |  |
| **Mesoscinus_managuae** | Mesoscincus_managuae |  |  |
| **Micrurus_annelatus** | Micrurus_corallinus |  | Slowinski 1995 |
| **Micrurus_nigrocinctus** | Micrurus_fulvius |  | Jowers et al. 2019 |
| **Micrurus_surinamensis** | Micrurus_surinamensis |  |  |
| **Nerodia_fasciata** | Nerodia_fasciata |  |  |
| **Nerodia_sipedon** | Nerodia_sipedon |  |  |
| **Nerodia_taxispilota** | Nerodia_taxispilota |  |  |
| **Ninia_maculata** | Ninia_atrata |  |  |
| **Ninia_sebae** | Ninia_atrata |  |  |
| **Ninia_sebai** | Ninia_atrata |  |  |
| **Nothopsis_rugosus** | Nothopsis_rugosus |  |  |
| **Opheodrys_aestivus** | Opheodrys_aestivus |  |  |
| **Opheodrys_ventralis** | Opheodrys_vernalis |  |  |
| **Oxybelis_aeneus** | Oxybelis_aeneus |  |  |
| **Oxybelis_fulgidus** | Oxybelis_fulgidus |  |  |
| **Oxyrhopus_formosus** | Oxyrhopus_formosus |  |  |
| **Oxyrhopus_melanogenys** | Oxyrhopus_melanogenys |  |  |
| **Pantherophis_alleghaniensis** | Pantherophis_alleghaniensis |  |  |
| **Pantherophis_guttatus** | Pantherophis_guttatus |  |  |
| **Phrynonax_poecilonotus** | Pseustes_poecilonotus |  |  |
| **Pituophis_catenifer** | Pituophis_catenifer |  |  |
| **Plestiodon_inexpectatus** | Plestiodon_inexpectatus |  |  |
| **Plica_plica** | Plica_plica |  |  |
| **Plica_umbra** | Plica_umbra |  |  |
| **Porthidium_nasutum** | Porthidium_nasutum |  |  |
| **Ptychoglossus_brevifrontalis** | Ptychoglossus_brevifrontalis |  |  |
| **Rhadinaea_kinkelini** | Rhadinaea_flavilata |  |  |
| **Rhinobothryum_bovalli** | Rhinobothryum_lentiginosum |  |  |
| **Rhinochylus_lecontei** | Rhinocheilus_lecontei |  |  |
| **Salvadora_hexalepis** | Salvadora_hexalepis |  |  |
| **Scaphiodontophis_annulatus** | Scaphiodontophis_annulatus |  |  |
| **Sceloporus_malachiticus** | Sceloporus_malachiticus |  |  |
| **Sceloporus_squamosus** | Sceloporus_squamosus |  |  |
| **Sceloporus_variabilis** | Sceloporus_variabilis |  |  |
| **Sibon_nebulatus** | Sibon_nebulatus |  |  |
| **Siphlopis_cervinus** | Siphlophis_cervinus |  |  |
| **Siphlopis_compressus** | Siphlophis_compressus |  |  |
| **Sistrurus_catenatus** | Sistrurus_catenatus |  |  |
| **Sphaerodactylus_millepunctatus** | Sphaerodactylus_millepunctatus |  |  |
| **Sphenomorphus_cherriei** | Scincella_cherriei |  |  |
| **Spilotes_pullatus** | Spilotes_pullatus |  |  |
| **Stenorrhina_degenharti** | Stenorrhina_freminvillei |  |  |
| **Storeria_dekayi** | Storeria_dekayi |  |  |
| **Storeria_occipitomaculata** | Storeria_occipitomaculata |  |  |
| **Taeniophallus_occipitalis** | Taeniophallus_affinis |  |  |
| **Tantilla_melanocephala** | Tantilla_vermiformis |  | Jowers et al. 2020 |
| **Thamnophis_butleri** | Thamnophis_butleri |  |  |
| **Thamnophis_sirtalis** | Thamnophis_sirtalis |  |  |
| **Thecadactylus_rapicauda** | Thecadactylus_rapicauda |  |  |
| **Thecadactylus_solimoensis** | Thecadactylus_solimoensis |  |  |
| **Trimorphodon_quadruplex** | Trimorphodon_biscutatus |  | Devitt 2003 |
| **Typhlops_reticulatus** | Amerotyphlops_reticulatus |  |  |
| **Urosaurs_ornatus** | Urosaurus_ornatus |  |  |
| **Uta_stansburiana** | Uta_stansburiana |  |  |
| **Xenopholis_scalaris** | Xenopholis_scalaris |  |  |
| **my species** | tonini |  |  |
| **Alopoglossus_angulatus** | Alopoglossus_angulatus |  |  |
| **Ameiva_ameiva** | Ameiva_ameiva |  |  |
| **Ameiva_festiva** | Holcosus_festivus |  |  |
| **Ameiva_undulata** | Holcosus_undulatus |  |  |
| **Anilius_scytale** | Anilius_scytale |  |  |
| **Anolis_biporcatus** | Anolis_biporcatus |  |  |
| **Anolis_capito** | Anolis_capito |  |  |
| **Anolis_carolinensis** | Anolis_carolinensis |  |  |
| **Anolis_cupreus** | Anolis_cupreus |  |  |
| **Anolis_dariensis** | Anolis_cupreus |  |  |
| **Anolis_fuscoauratus** | Anolis_fuscoauratus |  |  |
| **Anolis_lemurinus** | Anolis_lemurinus |  |  |
| **Anolis_limifrons** | Anolis_limifrons |  |  |
| **Anolis_nitens** | Anolis_chrysolepis |  |  |
| **Anolis_ortonii** | Anolis_ortonii |  |  |
| **Anolis_oxylophus** | Anolis_oxylophus |  |  |
| **Anolis_punctatus** | Anolis_punctatus |  |  |
| **Anolis_quaggulus** | Anolis_quaggulus |  |  |
| **Anolis_sagrei** | Anolis_sagrei |  |  |
| **Anolis_sericeus** | Anolis_sericeus |  |  |
| **Anolis_sp1** | Anolis_limifrons |  |  |
| **Anolis_tropidonotus** | Anolis_tropidonotus |  |  |
| **Aspidoscelis_deppii** | Aspidoscelis_deppei |  |  |
| **Aspidoscelis_motaguae** | Aspidoscelis_communis |  | Barley et al. 2019 |
| **Atractus_elaps** | Atractus_elaps |  |  |
| **Atractus_flammigerus** | Atractus_flammigerus |  |  |
| **Atractus_sp** | Atractus_flammigerus |  |  |
| **Atropoides_indomitus** | Atropoides_indomitus |  |  |
| **Bachia_dorbignyi** | Bachia_dorbignyi |  |  |
| **Bachia_trisanale** | Bachia_trisanale |  |  |
| **Basiliscus_plumifrons** | Basiliscus_plumifrons |  |  |
| **Basiliscus_vittatus** | Basiliscus_vittatus |  |  |
| **Bothriechis_schlegelii** | Bothriechis_schlegelii |  |  |
| **Bothriopsis_bilineata** | Bothrops_taeniata |  | Bernarde et al. 2021 |
| **Bothrops_asper** | Bothrops_asper |  |  |
| **Bothrops_brazili** | Bothrops_brazili |  |  |
| **Cercosaura_argulus** | Cercosaura_argulus |  |  |
| **Cercosaura_eigenmanni** | Cercosaura_eigenmanni |  |  |
| **Chironius_fuscus** | Chironius_fuscus |  |  |
| **Chironius_grandisquamis** | Chironius_grandisquamis |  |  |
| **Chironius_multiventris** | Chironius_multiventris |  |  |
| **Chironius_scurrulus** | Chironius_scurrulus |  |  |
| **Clelia_clelia** | Clelia_clelia |  |  |
| **Coleonyx_mitratus** | Coleonyx_mitratus |  |  |
| **Coluber_constrictor** | Coluber_constrictor |  |  |
| **Conophis_lineatus** | Conophis_lineatus |  |  |
| **Corallus_hortulanus** | Corallus_hortulanus |  |  |
| **Corytophanes_cristatus** | Corytophanes_cristatus |  |  |
| **Crotalus_adamanteus** | Crotalus_adamanteus |  |  |
| **Crotalus_horridus** | Crotalus_horridus |  |  |
| **Dendrophidion_dendrophis** | Dendrophidion_dendrophis |  |  |
| **Dendrophidion_percarinatum** | Dendrophidion_percarinatum |  |  |
| **Diadophis_punctatus** | Diadophis_punctatus |  |  |
| **Dipsas_articulata** | Dipsas_articulata |  |  |
| **Dipsas_catesbyi** | Dipsas_catesbyi |  |  |
| **Drepanoides_anomalus** | Drepanoides_anomalus |  |  |
| **Drymarchon_melanurus** | Drymarchon_corais |  |  |
| **Drymobius_rhombifer** | Drymobius_rhombifer |  |  |
| **Drymoluber_dichrous** | Drymoluber_dichrous |  |  |
| **Enulius_flavitorques** | Xenodon_merremi |  | Zaher et al. 2009, Myers 2014 |
| **Enulius_sclateri** | Xenodon_merremi |  |  |
| **Epicrates_cenchria** | Epicrates_cenchria |  |  |
| **Erythrolamprus_sp** | Erythrolamprus_reginae |  | co-occurs |
| **Gonatodes_albogularis** | Gonatodes_albogularis |  |  |
| **Gonatodes_hasemani** | Gonatodes_hasemani |  |  |
| **Gonatodes_humeralis** | Gonatodes_humeralis |  |  |
| **Gymnopthalmus_speciosus** | Gymnophthalmus_speciosus |  |  |
| **Helicops_angulatus** | Helicops_angulatus |  |  |
| **Heterodon_nasicus** | Heterodon_nasicus |  |  |
| **Imantodes_cenchoa** | Imantodes_cenchoa |  |  |
| **Imantodes_chenchoa** | Imantodes_cenchoa |  |  |
| **Imantodes_lentiferus** | Imantodes_lentiferus |  |  |
| **Iphisa_elegans** | Iphisa_elegans |  |  |
| **Kentropyx_pelviceps** | Kentropyx_pelviceps |  |  |
| **Lampropeltis_abnorma** | Lampropeltis_abnorma |  |  |
| **Lampropeltis_getula** | Lampropeltis_getula |  |  |
| **Lepidophyma_flavimaculatum** | Lepidophyma_flavimaculatum |  |  |
| **Leptodeira_annulata** | Leptodeira_annulata |  |  |
| **Leptodeira_nigrofasciata** | Leptodeira_nigrofasciata |  |  |
| **Leptodeira_rhombifera** | Leptodeira_annulata |  | Barrio-Amoros et al. 2019 |
| **Leptodeira_septentrionalis** | Leptodeira_septentrionalis |  |  |
| **Leptodrymus_pulcherrimus** | Leptodrymus_pulcherrimus |  |  |
| **Leptophis _ahaetulla** | Leptophis_ahaetulla |  |  |
| **Masticophis_flagellum** | Coluber_flagellum |  |  |
| **Mastigodryas_alternatus** | Mastigodryas_melanolomus |  | Montingelli et al. 2019 |
| **Mesaspis_moreletti** | Mesaspis_gadovii |  |  |
| **Mesoscinus_managuae** | Mesoscincus_managuae |  |  |
| **Micrurus_annelatus** | Micrurus_corallinus |  | Slowinski 1995 |
| **Micrurus_nigrocinctus** | Micrurus_fulvius |  | Jowers et al. 2019 |
| **Micrurus_surinamensis** | Micrurus_surinamensis |  |  |
| **Nerodia_fasciata** | Nerodia_fasciata |  |  |
| **Nerodia_sipedon** | Nerodia_sipedon |  |  |
| **Nerodia_taxispilota** | Nerodia_taxispilota |  |  |
| **Ninia_maculata** | Ninia_atrata |  |  |
| **Ninia_sebae** | Ninia_atrata |  |  |
| **Ninia_sebai** | Ninia_atrata |  |  |
| **Nothopsis_rugosus** | Nothopsis_rugosus |  |  |
| **Opheodrys_aestivus** | Opheodrys_aestivus |  |  |
| **Opheodrys_ventralis** | Opheodrys_vernalis |  |  |
| **Oxybelis_aeneus** | Oxybelis_aeneus |  |  |
| **Oxybelis_fulgidus** | Oxybelis_fulgidus |  |  |
| **Oxyrhopus_formosus** | Oxyrhopus_formosus |  |  |
| **Oxyrhopus_melanogenys** | Oxyrhopus_melanogenys |  |  |
| **Pantherophis_alleghaniensis** | Pantherophis_alleghaniensis |  |  |
| **Pantherophis_guttatus** | Pantherophis_guttatus |  |  |
| **Phrynonax_poecilonotus** | Pseustes_poecilonotus |  |  |
| **Pituophis_catenifer** | Pituophis_catenifer |  |  |
| **Plestiodon_inexpectatus** | Plestiodon_inexpectatus |  |  |
| **Plica_plica** | Plica_plica |  |  |
| **Plica_umbra** | Plica_umbra |  |  |
| **Porthidium_nasutum** | Porthidium_nasutum |  |  |
| **Ptychoglossus_brevifrontalis** | Ptychoglossus_brevifrontalis |  |  |
| **Rhadinaea_kinkelini** | Rhadinaea_flavilata |  |  |
| **Rhinobothryum_bovalli** | Rhinobothryum_lentiginosum |  |  |
| **Rhinochylus_lecontei** | Rhinocheilus_lecontei |  |  |
| **Salvadora_hexalepis** | Salvadora_hexalepis |  |  |
| **Scaphiodontophis_annulatus** | Scaphiodontophis_annulatus |  |  |
| **Sceloporus_malachiticus** | Sceloporus_malachiticus |  |  |
| **Sceloporus_squamosus** | Sceloporus_squamosus |  |  |
| **Sceloporus_variabilis** | Sceloporus_variabilis |  |  |
| **Sibon_nebulatus** | Sibon_nebulatus |  |  |
| **Siphlopis_cervinus** | Siphlophis_cervinus |  |  |
| **Siphlopis_compressus** | Siphlophis_compressus |  |  |
| **Sistrurus_catenatus** | Sistrurus_catenatus |  |  |
| **Sphaerodactylus_millepunctatus** | Sphaerodactylus_millepunctatus |  |  |
| **Sphenomorphus_cherriei** | Scincella_cherriei |  |  |
| **Spilotes_pullatus** | Spilotes_pullatus |  |  |
| **Stenorrhina_degenharti** | Stenorrhina_freminvillei |  |  |
| **Storeria_dekayi** | Storeria_dekayi |  |  |
| **Storeria_occipitomaculata** | Storeria_occipitomaculata |  |  |
| **Taeniophallus_occipitalis** | Taeniophallus_affinis |  |  |
| **Tantilla_melanocephala** | Tantilla_vermiformis |  | Jowers et al. 2020 |
| **Thamnophis_butleri** | Thamnophis_butleri |  |  |
| **Thamnophis_sirtalis** | Thamnophis_sirtalis |  |  |
| **Thecadactylus_rapicauda** | Thecadactylus_rapicauda |  |  |
| **Thecadactylus_solimoensis** | Thecadactylus_solimoensis |  |  |
| **Trimorphodon_quadruplex** | Trimorphodon_biscutatus |  | Devitt 2003 |
| **Typhlops_reticulatus** | Amerotyphlops_reticulatus |  |  |
| **Urosaurs_ornatus** | Urosaurus_ornatus |  |  |
| **Uta_stansburiana** | Uta_stansburiana |  |  |
| **Xenopholis_scalaris** | Xenopholis_scalaris |  |  |
